# Interactive workshops on improving dental students’ understanding of environmentally sustainable dentistry: a quality improvement project

**DOI:** 10.1038/s41405-026-00427-y

**Published:** 2026-04-16

**Authors:** Florence Mai, Edward Parkinson, Fleur Mumford, Zahra Shehabi

**Affiliations:** 1 Institute of Dentistry, Queen Mary University of London, Barts and the London School of Medicine and Dentistry Dental Hospital, London, UK; 2 NHS Workforce, Training and Education (WTandE), London, UK; 3 Barts NHS Health Trust, Institute of Dentistry, The Royal London Dental Hospital, Queen Mary University of London, Barts and the London School of Medicine and Dentistry Dental Hospital, London, UK

**Keywords:** Continuing professional development in dentistry, Dental clinical teaching

## Abstract

**Background:**

Environmental sustainability in dentistry (ESD) is increasingly recognised by major organisations as essential, yet teaching of it remains fragmented and inconsistent across UK dental schools. This project evaluates teaching designed to enhance students’ confidence and knowledge of ESD.

**Methods:**

A two-part interactive workshop series was delivered to dental and dental hygiene and therapy students at Queen Mary University London. Workshop 1 combined didactic lectures with group-based brainstorming of sustainability challenges. Workshop 2 centred on student-led presentations proposing responses to challenges for stakeholders. Pre- and post-workshop surveys assessed confidence in four domains with a 10-point Likert scale, and knowledge with six multiple-choice questions.

**Results:**

Confidence scores increased across domains and knowledge scores improved following the intervention. Participants rated the workshops highly on usefulness and enjoyment. Feedback identified the need to improve workshop timing and reduce assignment workload.

**Discussion:**

Low baseline confidence and knowledge scores indicated a need to incorporate ESD into the curriculum. The mixed-method approach was associated with positive change, with one proposal progressing toward real-world implementation.

**Conclusion:**

Workshop-based teaching was associated with improved student confidence and knowledge in ESD. Early curriculum integration is recommended, aligning with the 2025 GDC Safe Practitioner Framework requirements for ESD education.

## Background

Healthcare delivery is currently environmentally, socially and financially unsustainable [1]. The healthcare sector accounts for 5% of global greenhouse gas emissions, with oral healthcare emerging as a notable contributor [2]. National and international organisations have acknowledged the contribution of the wider healthcare sector to climate change through multiple policy documents such as the Brundtland report, the Paris Agreement, UN Sustainable Development Goals, COP reports and the ‘Delivering Net Zero NHS’ report of NHS England [3–7]. The oral health sector is a notable contributor to climate change through the generation of waste, pollution and greenhouse gas emissions contributing to environmental pressures that have been described as a ‘fundamental threat’ to human health [3, 8]. Patient and staff travel, procurement and energy use are the major carbon hotspots in dentistry [9, 10], and it is estimated that a typical UK dental practice contributes approximately 675 kg CO₂ equivalents per dentist per month [11].

Embedding environmental sustainability in dentistry (ESD) in education has therefore been highlighted as a key strategy to mitigate the environmental impacts of dentistry on our environment [11, 12]. Effective ESD education should begin at the undergraduate level and continue throughout a dental professional’s career [13]. Dental students themselves recognise the importance of sustained engagement with sustainability across their working lives to mitigate the environmental consequences of clinical practice [2].

The Association for Dental Education in Europe (ADEE) aided in the creation of the “Graduating European Dentist” curricula, which were created to outline how to teach ESD to undergraduates. They further published two consensus reports highlighting the learning outcomes for teaching and examination of ESD [14, 15]. Furthermore, the GDC updated its safe practitioner framework to include ESD as part of undergraduate, postgraduate and continued professional development teaching [16], aligning with the World Dental Federation’s (FDI) ‘Sustainable Dentistry Toolkit’ [17].

Despite this, recent studies show that integration of ESD into the curricula remains sparse, inconsistent and underdeveloped [11]. Dixon (2024) found that 56% of dental schools based in the UK and Ireland do not teach ESD [18], with only 32.4% of European dental institutions planning to integrate ESD into their curricula [19]. A scoping review examining the integration of ESD into teaching practices identified several key challenges, including gaps in policy frameworks, limitations in faculty development programmes (including lack of time, funding and resources), and the need for more innovative teaching strategies [11, 18]. Furthermore, key barriers include that [1] the dental curriculum is already overloaded and [2] the lack of knowledge to teach ESD [18]. Existing teaching strategies of key topics such as ESD include mainly practical exercises and didactic delivery, with less focus on flipped classroom and problem-based learning [19]. Dixon (2025) further highlighted the importance of longitudinal embedding of undergraduate ESD teaching from year 1 to year 5 as the most effective way to teach ESD [19]. Despite increasing policy emphasis and emerging curricular guidance, empirically evaluated educational interventions in undergraduate dentistry remain limited [20].

A cross-sectional survey for students and educators at Queen Mary University of London (QMUL) and Harvard School of Dental Medicine found that neither school had formal ESD teaching [21]. We consider this quality improvement project to be timely, addressing a gap in the formal teaching of ESD and utilising an innovative approach. This quasi-experimental study therefore evaluates a two-part interactive workshop series designed to explore improvements in dental students’ knowledge, confidence, and engagement with ESD.

## Methodology

### Study design

There is a growing body of evidence indicating that interactive teaching approaches are more effective than didactic methods alone in promoting behaviour change and achieving meaningful improvements in professional practice, particularly within medical education [22]. In addition, a 2021 Cochrane review highlighted that combining multiple teaching methods or behaviour change techniques can address different learning styles, supporting the use of a balanced approach incorporating both interactive and passive elements [23]. Drawing on this evidence, alongside previous local successes with workshop-based teaching [24], a novel two-part interactive workshop series was developed for delivery within the clinical years of the BDS and BScOH curriculum. This study was designed as a quasi-experimental pre–post quality improvement evaluation using independent samples and was not intended to support causal inference.

The design of the workshops was underpinned by Biggs’ constructive alignment model [25], in which students actively constructed their knowledge through engagement with the workshop framework, which was aligned with specific learning outcomes (see Table 1) and the final assessed presentations. Within this framework, the educator acted as a facilitator. When considering assessment formats, priority was given to creating an authentic assessment [26]. The final student presentation reflected tasks students may encounter after graduation. The use of video presentations further supported engagement, accessibility of information, and meaningful knowledge transfer.

**Table 1:** Learning objectives and outcomes.

	Learning objectives	Learning outcomes
Workshop 1	Introduce core principles of environmental sustainability within dentistry and healthcare	Students demonstrate understanding of sustainability principles and their relevance to clinical dentistry
	Develop students’ understanding of dentistry’s environmental footprint and opportunities for improvement	Students can describe key sources of environmental impact within dental practice and education
	Introduce foundational concepts of innovation and project management relevant to sustainability initiatives, such as the triple bottom line framework	Students can identify and plan feasible sustainability challenges within dental education or practice
	Encourage collaborative, student-centred learning through active engagement with a sustainability challenge	Students work collaboratively to develop an initial sustainability project proposal using basic innovation and project planning concepts
Workshop 2	Provide students with an opportunity to apply learning through project development and presentation	Students present a structured sustainability proposal clearly and confidently using a video-based format
	Build confidence in communicating sustainability ideas effectively to academic and clinical stakeholders	Students justify proposed interventions with consideration of both environmental impact and practical implementation
	Encourage critical evaluation of sustainability initiatives based on impact, feasibility, and implementation	Students critically evaluate their own and peers’ projects
	Promote engagement with sustainability stakeholders and real-world application of student ideas	Students demonstrate increased confidence in advocating for sustainable practices within dental education and future clinical practice

The first workshop series was piloted in 2023 with a small focus group of 10 volunteer dental student participants (fourth- and fifth-year BDS students). The workshop series was subsequently refined in response to feedback from the pilot and was then implemented in 2025 as part of the formal teaching programme. QMUL identifies ‘promoting sustainability’ as a core graduate attribute, requiring students to demonstrate an understanding of sustainability within their discipline [26]; these workshops provided a structured opportunity for students to evidence and develop this competency. The effectiveness of the workshop series was evaluated using pre- and post-workshop questionnaires, which were used to assess changes in confidence and knowledge across the series, as well as to collect both qualitative and quantitative feedback.

### Intervention

**Fig. 1:**
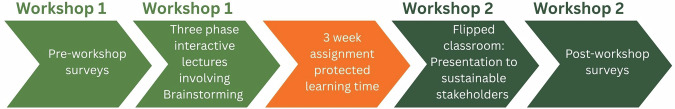
Structure of the two-part workshop series.

**Fig. 2:**
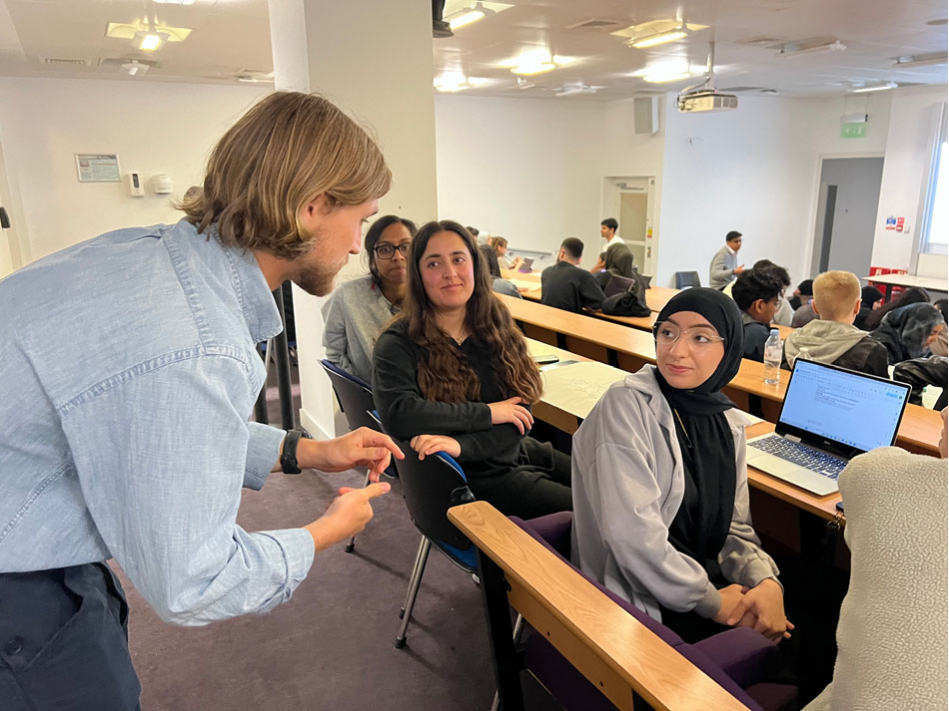
Phase 1 presentations, Workshop 1.

**Fig. 3:**
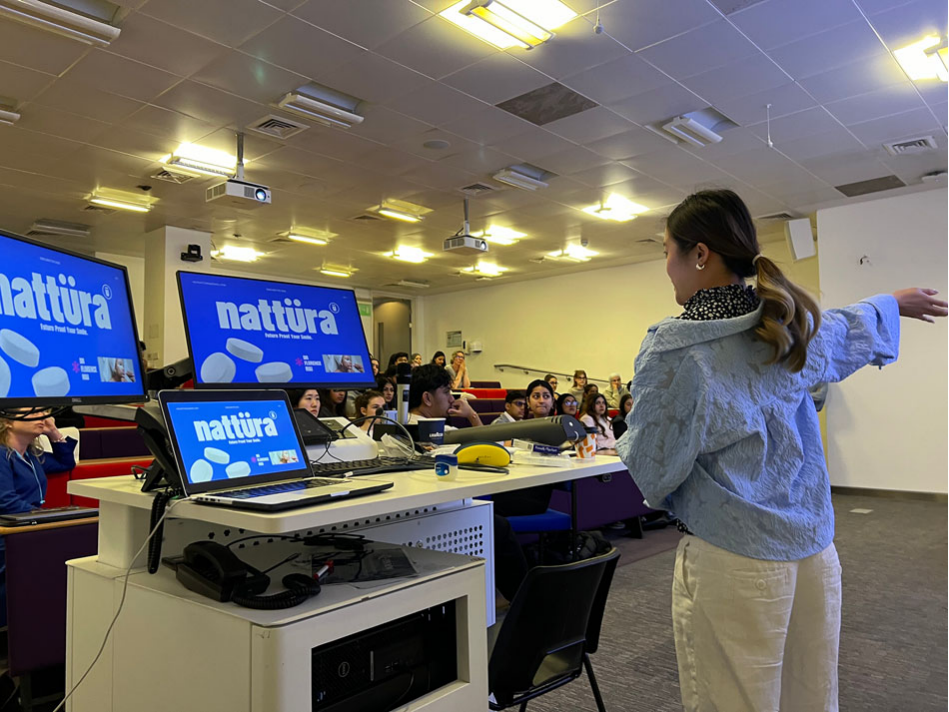
Phase 2 presentation, Workshop 1.

**Fig. 4:**
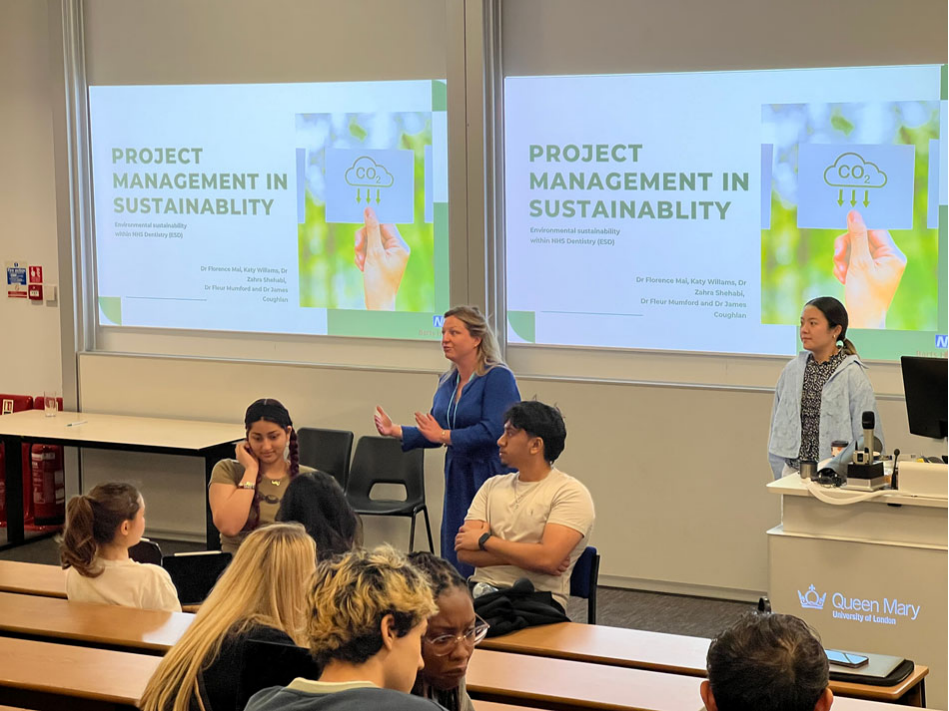
Phase 3 brainstorming, Workshop 1.

Figure 1 shows the structure of the workshop series delivery. Each session lasted 3–4 h and was targeted to the whole of the third-year dental student and second-year dental hygiene and therapy year groups. Workshop 1 was delivered on 28/05/2025, with 44 students in attendance (45% attendance), and the student presentations (workshop 2) were delivered 3 weeks later on 18/06/2025, with 91 students in attendance (93% attendance). Both workshops were delivered in person and recorded to accommodate students unable to attend and to allow all participants to review the lectures online in their own time. It was determined that a three-week interval was sufficient to enable participants to undertake independent research and prepare their proposals while remaining short enough to maintain engagement and prevent loss of momentum amid competing academic commitments. To promote student engagement, prize incentives were used for the presentations, sponsored by SURI (sustainable toothbrush manufacturer) (Figs. 2–4).

Workshop 1: Foundations of Sustainability and Project Development

Workshop 1 was structured into 3 phases [1]: Understanding Environmentally Sustainable Dentistry and Healthcare’s Carbon Footprint [2], From University Project to Dental Start-Up: A Project Management Case Study and [3] Sustainability Challenge Dental Projects: From Concept to Proposal.

In phase 1, an introductory presentation on climate change and its impacts on dentistry within healthcare was given. Sustainability principles, including the triple bottom line, were outlined, and several real-world climate impact cases were presented, including local examples such as the Guy’s Hospital data centre overheating during the July 2022 heatwave. In phase 2, a former student and core researcher (FM) presented her university-funded toothpaste tablet start-up project, providing a map and inspiration for phase 3. In phase 3, students were divided into their ten clinical groups, each allocated a sustainability challenge: laboratory waste, reusable kits, eco-awareness campaigns, active travel, meat-free initiatives, PPE reduction, energy saving, wastewater management, carbon offsetting and AI-driven oral health mapping. Students were supported by a team of educators made up of clinical lecturers, industry and healthcare professionals and recent graduates; they helped facilitate initial brainstorming sessions, helping students to refine their ideas and guiding them through practical considerations, including stakeholder engagement, financial feasibility, patient safety, and infection prevention and control. Following this, many educators remained available for further guidance throughout the subsequent three weeks of protected study time and helped link groups with sustainability experts and relevant hospital staff. This enabled students to develop realistic, implementable proposals informed by real-world clinical and organisational perspectives.

### Workshop 2: Student-led Presentations

The ten groups each presented their dental sustainability solution to a judging panel of sustainability experts and senior NHS clinicians. Proposals were evaluated against six predefined criteria: clarity of issue and solution, creativity, feasibility, environmental impact, audience engagement, and stakeholder input. Each group was then given a score on a 10-point Likert scale (1=poor and 10=excellent) based on these criteria and ranked. The three highest-scoring groups were announced at the end of the session, and the highest-scoring group members were awarded a SURI sustainable electric toothbrush. The depth and quality of the student projects were impressive, with several groups producing highly detailed and well-presented proposals. The winning group focused on the introduction of tap aerators, providing an impressive level of analysis that included cost–benefit modelling and stakeholder involvement. Their work particularly impressed a member of the judging panel from the Trust’s sustainability team, who offered to support the group in taking the idea forward. Since then, the students have engaged directly with the sustainability team and presented their video to council members at the Tower Hamlets Borough Climate Partners’ meeting.

### Pre- and post-workshop surveys

Table 2 outlines the qualitative and quantitative questions included in the pre- and post-workshop surveys. These were developed by the authors (FM, EP, ZS and FM) and informed by the workshop learning objectives, relevant policy frameworks, and existing literature on environmental sustainability in dentistry. The confidence questions used a Likert scale of 1-10 (1 - not confident, 10 - very confident). The knowledge questions were multiple choice, giving the participants one correct answer and three incorrect answers. Content validity was supported through internal peer review by academic and clinical staff involved in sustainability teaching. The questionnaire was not formally piloted prior to delivery. Internal consistency of the instrument was not assessed, as the survey was designed as a pragmatic evaluation tool within a quality improvement framework rather than a psychometric measure.

**Table 2:** Pre- and post-workshop survey questions.

Pre-workshop survey questions	Post-workshop survey questions
What year of BDS are you?	
Do you study BDS as an undergraduate or postgraduate?	
How confident do you feel in identifying areas within your dental practice where environmental impact can be reduced? Scale 1-10 (1 - not confident, 10 - very confident)	
How confident do you feel to discuss and advocate for sustainable practices with colleagues and patients? Scale 1-10 (1 - not confident, 10 - very confident)	
How confident are you in implementing dental sustainability initiatives in your work environment (e.g., proper waste segregation, reducing single-use plastics, promoting active travel)? Scale 1-10 (1 - not confident, 10 - very confident)	
How confident do you feel in balancing environmental sustainability with maintaining high-quality patient care? Scale 1-10 (1 - not confident, 10 - very confident)	
Which of the following contributes the most to the carbon footprint of NHS dentistry? (Marked as correct or incorrect)	
The ‘Triple Bottom Line’ framework assesses sustainability based on which three key values? (Marked as correct or incorrect)	
Which waste stream has the most significant environmental impact in dentistry? (Marked as correct or incorrect)	
Which single dental procedure has the highest carbon footprint? (Marked as correct or incorrect)	
Which policy emphasises the duty to protect the health of current and future generations by reducing greenhouse gas emissions? (Marked as correct or incorrect)	
What percentage of global greenhouse gas emissions is attributed to the healthcare sector? (Marked as correct or incorrect)	
	Rate the usefulness of the presentations/lectures in workshop 1. Scale 1-10 (1 - not useful, 10 - very useful)
	Rate the usefulness of the discussions/brainstorming for the assignment in workshop 1. Scale 1-10 (1 - not useful, 10 - very useful)
	Rate how much you enjoyed workshop 1. Scale 1-10 (1 - not enjoyable, 10 - very enjoyable)
	Do you have any suggestions for improvements for workshop 1?
	Rate the usefulness of seeing other groups’ presentations in workshop 2. Scale 1-10 (1 - not useful, 10 - very useful)
	Rate how much you enjoyed presenting at the second workshop. Scale 1-10 (1 - not enjoyable, 10 - very enjoyable)
	Rate the usefulness of the feedback from the panel after your presentation in workshop 2. Scale 1-10 (1 - not useful, 10 - very useful)
	Do you have any suggestions for improvements for workshop 2?
	Rate how useful you thought the assignment was towards learning about dental sustainability. Scale 1-10 (1 - not useful, 10 - very useful)
	Rate how much you enjoyed working on your assignment. Scale 1-10 (1 - not enjoyable, 10 - very enjoyable)
	Rate how well you think your team performed. Scale 1-10 (1 - not good, 10 - very good)
	Do you have any suggestions for improvements for the assignment?
	Rate the teaching style for environmental sustainability in dentistry Scale 1-10 (1 - not good, 10 - very good)
	Which presentation did you enjoy the most?
	Which project do you think was the best?

Questions were repeated to monitor changes in confidence and knowledge.

We measured two key indicators: confidence and knowledge. These were used to evaluate the effectiveness of the intervention. The surveys were distributed to students through a QR code displayed during the workshops. Due to the anonymous survey design, responses were not matched to individual participants; therefore, pre- and post-workshop data were analysed as independent samples rather than paired comparisons. Quantitative confidence data were analysed using independent-samples t-tests in Microsoft Excel, and figures presented in Canva. Means and standard deviations were calculated for each confidence domain at pre- and post-workshop time points. Exact *p*-values and effect sizes (Cohen’s d) were reported where appropriate. Knowledge outcomes were analysed descriptively due to the limited number of items.

### Participant selection

This was conducted among third-year dental students and second-year dental hygiene and therapy students. Third-year students were chosen with convenience non-probability sampling, as they are at the optimum time for this learning before entering clinical practice. Behaviours learnt during such contextual transitions will be sustained, potentially establishing environmentally sustainable practices throughout their careers [27]. The workshop was a formative aspect of the curriculum; therefore, it was officially timetabled and attendance was mandatory. Written consent for participation was taken in both the pre- and post- workshop survey. Consent was also implied by completing the voluntary, anonymous survey. Ten expert panellists to judge student presentations were recruited over email and involved experts in dental environmental sustainability, public health and dental business strategy. Findings are therefore interpreted as associations with change rather than evidence of definitive intervention effects.

## Results

### Quantitative results

#### Survey response rate

Survey responses were analysed on the assumption that the total eligible participant pool comprised the combined cohort of dental and dental hygiene and therapy students (*n* = 98). As the sessions were recorded and uploaded for online access, the QR code linking to the pre- and post-workshop questionnaires was accessible to all students, including those unable to attend in person. Consequently, questionnaire completion was not limited to physical attendance at the workshops. In Workshop 1, 44 of 98 students completed the pre-workshop questionnaire (response rate 45%). Following Workshop 2, 91 of 98 students completed the post-workshop questionnaire (response rate 93%). All response rates and percentage changes were therefore calculated using the total cohort size as the denominator, reflecting both in-person and asynchronous engagement with the teaching. The differing number of responses between the sessions should be considered when interpreting findings. As analyses were conducted using independent samples, observed changes reflect group-level trends rather than within-participant change.

#### Workshop participant demographics

Overall, 44 participants attended the first workshop, and 91 participants attended the second workshop. Demographic data were only gathered in the first workshop; this found 81.4% (*n* = 36) were third-year students and 18.2% (*n* = 8) were second-year hygiene and therapy students. Most students, 84.1% (*n* = 37), were studying dentistry as an undergraduate degree, having not completed a degree before, and 15.9% (*n* = 7) were studying dentistry as a postgraduate degree, having completed a prior degree or degrees, i.e., graduate entry.

### Summary of quantitative results

**Fig. 5:**
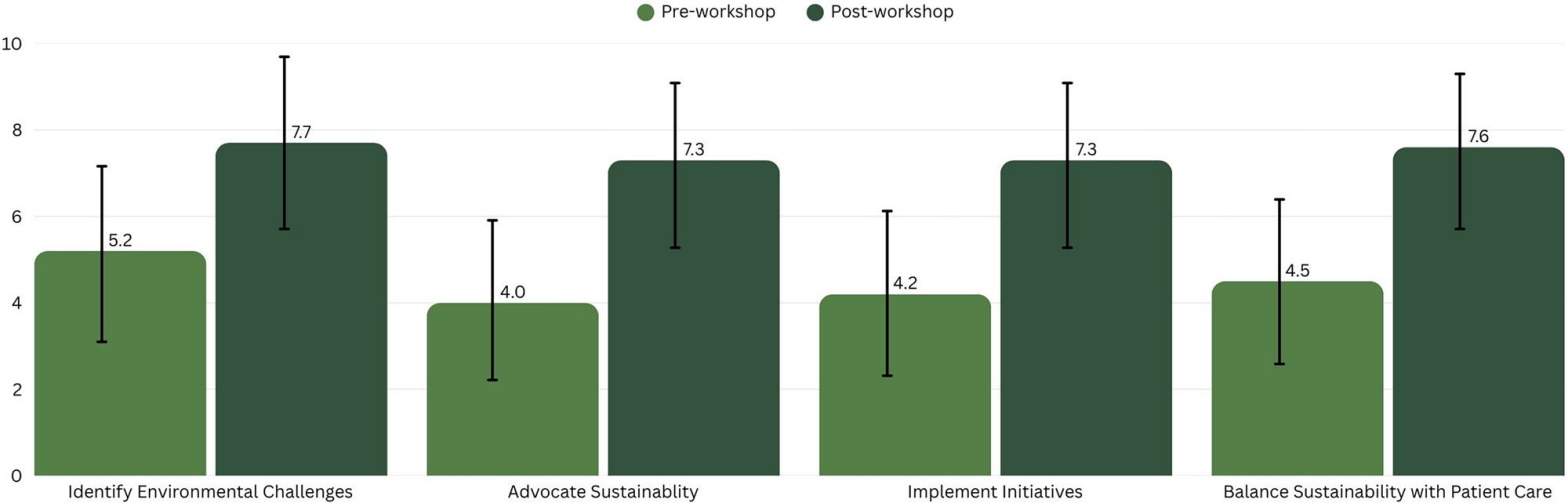
Average participant confidence pre- and post-workshop.

Mean confidence scores increased across all domains from pre- to post-workshop (Fig. 5). Confidence in identifying environmental impact increased from 5.16 ± 2.05 to 7.71 ± 1.96 (49% increase), advocating sustainability from 3.98 ± 1.87 to 7.31 ± 1.85 (84% increase), implementing sustainability initiatives from 4.18 ± 1.88 to 7.29 ± 1.88 (74% increase), and balancing sustainability with patient care from 4.45 ± 1.86 to 7.63 ± 1.71 (71% increase). Improvements were statistically significant across domains (exact *p* < 0.05), with large effect sizes (Cohen’s d ranging from 1.27 to 1.79).

**Fig. 6:**
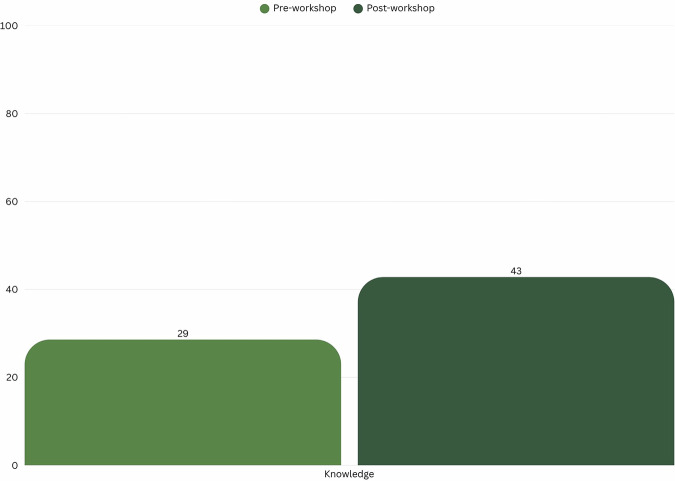
Average participant knowledge pre- and post-workshop.

Knowledge scores increased from 29% to 43% correct responses (48% relative increase). Knowledge outcomes were analysed descriptively due to the limited number of items and therefore no statistical testing was conducted (Fig. 6).

### Qualitative results

**Fig. 7:**
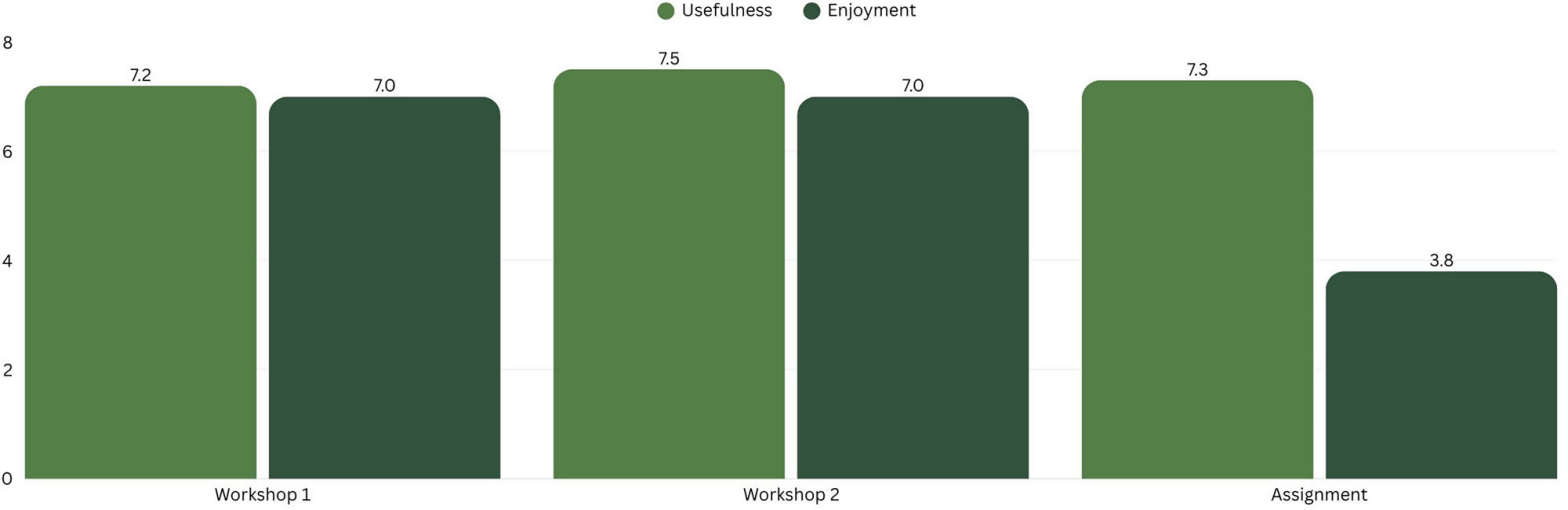
Average participant scores on usefulness and enjoyment of each aspect of the workshop series.

Participants rated Workshop 1, Workshop 2 and the assignment highly on usefulness towards learning ESD. They scored an average usefulness score out of 10 with Workshop 1 (7.2), Workshop 2 (7.5) and the assignment (7.3). Participants further rated the enjoyment, where they scored an average enjoyment score out of 10 with Workshop 1 (7.0), Workshop 2 (7.0) and the assignment (3.8). The assignment scored low on enjoyment. This is illustrated in Fig. 7. Participants were further asked if they had any suggestions on improvements for each aspect of the workshop. Selected suggestions that best represent the feedback are shown in Table 3.

**Table 3:** Summarised feedback from students on each aspect of the workshop series.

Workshop aspect	Feedback summary
Workshop 1	‘Students wanted more time to prepare’ ‘More structured and meaningful brainstorming’ ‘Timetable clash with this assignment and other assignments’
Workshop 2	‘Shorten presentation timings’ ‘Halving the year group and running two sessions to improve engagement’ ‘Providing written feedback after the assignment is finished’
Assignment	‘More interactive Q&A’ ‘Shorter presentation to smaller audiences’ ‘Clearer student voice and post-event feedback from judges’

## Discussion

The low baseline confidence and knowledge among students regarding ESD demonstrates that this topic has not yet been effectively integrated into the curriculum. This result aligns with existing literature, which highlights the inconsistent and fragmented nature of sustainability teaching [9], which varies globally between institutions. As indicated by previous literature and a Cochrane review [23], workshop-based teaching methods that incorporate both active/interactive and passive/didactic learning may be key to introducing ESD into the undergraduate curricula as a new subject.

This study targeted both confidence and knowledge. Confidence was assessed across four domains: identifying environmental challenges, advocating sustainability, implementing environmental initiatives and balancing sustainability with patient care. Knowledge was assessed with a mini quiz of six questions that increased in perceived difficulty determined by the research team. However, the number of questions for knowledge may have been too little to make a conclusive judgement about knowledge increase. The researchers would recommend wider summative testing.

Although knowledge scores improved following the intervention, the absolute post-workshop score suggests partial understanding rather than full competency. This could indicate that while short-term gains were observed, a single workshop series is unlikely to be educationally sufficient and that sustainability education may require longitudinal integration across the curriculum to support deeper understanding. In addition, short-term improvements in knowledge do not necessarily translate into sustained retention or behavioural change, highlighting the importance of ongoing reinforcement and future evaluation of longer-term outcomes. Importantly, sustainability competencies extend beyond knowledge acquisition alone and include a broader range of professional skills, such as communication, leadership, and collaborative problem-solving, as reflected in emerging curriculum frameworks [14]. The workshop format appeared to support development across these broader domains through applied, student-led project work[28].

Improvements in confidence may also be interpreted through a self-efficacy lens, whereby increased perceived capability is associated with greater engagement, effort and persistence in performance [29]. In this context, opportunities for active participation, collaboration, and presentation may have contributed to strengthening students’ confidence in applying sustainability principles. These findings can also be considered within behaviour change frameworks such as the capability–opportunity–motivation (COM-B) model [30]. The workshops may have enhanced capability through knowledge development, opportunity through collaborative learning and stakeholder engagement, and motivation through authentic assessment and peer interaction.

A key methodological consideration relates to the difference in participant numbers between pre- and post-workshop responses. As responses were anonymous and analysed using independent samples, observed changes cannot be interpreted as within-participant effects. Although both workshops were delivered to the same student cohort, the degree of overlap between respondents cannot be confirmed. It is likely that many students who attended Workshop 2 also attended Workshop 1 as they were mandatory workshops. However, the post-workshop sample may also include students not represented in the pre-workshop sample, potentially inflating or altering observed differences. The larger post-workshop sample may also have influenced effect estimation by introducing additional respondents who were not represented at baseline. In addition, response bias may have influenced findings if more engaged or motivated students were more likely to complete the post-workshop survey. Consequently, results should be interpreted cautiously as indicative group-level associations rather than definitive intervention effects, although consistent trends across domains suggest favourable cohort-level shifts in knowledge and confidence.

The inclusion of video presentations appeared to be a particularly effective element of the intervention, producing high-quality videos that engaged judges and other sustainability stakeholders. Although students reported high levels of enjoyment for both workshops, enjoyment of the assignment was rated lower, which may reflect the additional time commitment and demands associated with developing and presenting a project-based proposal alongside existing academic workloads.

Feedback identified areas for refinement, including reducing class sizes and re-optimising assignment workload, essential for long-term adoption. These findings support the need for iterative and scaffolded approaches to sustainability education rather than reliance on single interventions. Integration of ESD into undergraduate curricula, aligned with the GDC 2025 update on the safe practitioner framework [7], may foster environmentally conscious behaviours early in training and prepares graduates for climate-conscious practice.

### Limitations

This study reports descriptive statistics for the pre-workshop and post-workshop survey; therefore, findings should be interpreted cautiously. Responses were anonymous and could not be paired across time points, preventing within-participant comparisons. Analyses were conducted using independent samples, and differences in response numbers between workshops may reflect variation in attendance, engagement, or survey accessibility. Consequently, observed changes cannot be interpreted as individual-level effects and should instead be considered indicative of cohort-level trends. While this limits internal validity, the findings remain useful for informing educational design and future research. Demographic data beyond programme year were not collected; therefore, subgroup analyses were not undertaken and participant diversity cannot be assessed.

The survey instrument was developed for this study and was not externally validated. The limited number of knowledge items may restrict sensitivity in detecting changes and reduce reliability. In addition, internal consistency was not formally assessed, which may limit interpretation of the precision of knowledge and confidence measures.

Attendance also represents a limitation. Workshop 1 achieved 45% attendance despite being mandatory and formally recorded. Reduced attendance at in-person teaching has been widely reported across UK universities following the COVID-19 pandemic, and this trend has been similarly observed at QMUL. Targeted communication was sent to students between the two workshops to emphasise the importance of participation, which may have contributed to improved attendance at Workshop 2. Recorded delivery and group-based project work allowed non-attendees to access material and contribute indirectly; however, variation in attendance may have influenced the representativeness of responses.

The paper evaluated short-term outcomes only; therefore, sustained retention and longer-term behavioural impact could not be assessed. Although the intervention facilitated subsequent engagement with sustainability initiatives for some students, these outcomes were not systematically evaluated and cannot be interpreted as evidence of sustained behavioural change.

## Conclusion

This study suggests that workshop-based mixed-method learning may support the development of environmentally conscious behaviours in dental/dental hygiene and therapy students. These findings support consideration of early curriculum integration of undergraduate training to foster climate-conscious behaviours for the dental careers ahead. Further research using longitudinal and controlled study designs is needed to determine sustained impact and causal effects.

## Data Availability

Data is available upon reasonable request from the authors.

## References

[1] Duane B, Stancliffe R, Miller FA, Sherman J, Pasdeki-Clewer E. Sustainability in dentistry: a multifaceted approach needed. J Dent Res. 2020;99:998–1003. 32392435 10.1177/0022034520919391

[2] Durnall O, Martin N, Mulligan S, Dixon J. Environmental sustainability: the attitudes and experiences of UK students in the oral health care profession. Br Dent J. 2024. 10.1038/s41415-024-7135-z. 10.1038/s41415-024-7135-z 38443612

[3] Field J, Martin N, Duane B, Vital S, Mulligan S, Livny A, et al. Embedding environmental sustainability within oral health professional curricula-Recommendations for teaching and assessment of learning outcomes. Eur J Dent Educ. 2023;27:650–61. 36121067 10.1111/eje.12852

[4] World Commission on Environment and Development. Our Common Future (The Brundtland Report). Oxford: Oxford University Press; 1987.

[5] UNFCCC. Adoption of the Paris Agreement. UN Doc FCCC/CP/2015/L.9/Rev.1. Paris: UNFCCC; 2015. Available at: https://unfccc.int/resource/docs/2015/cop21/eng/l09r01.pdf.

[6] UNFCCC. COP28 UAE Declaration on Climate and Health. Dubai: UNFCCC; 2023. Available at: https://cdn.who.int/media/docs/default-source/climate-change/cop28/cop28-uae-climate-and-health-declaration.pdf.

[7] NHS England. Delivering a Net Zero National Health Service. London: NHS England; 2020. Available at: https://www.england.nhs.uk/greenernhs/publication/delivering-a-net-zero-national-health-service/.

[8] Forzieri G, Cescatti A, E Silva FB, Feyen L. Increasing risk over time of weather-related hazards to the European population: a data-driven prognostic study. Lancet Planet Health. 2017;1:e200-8. 10.1016/S2542-5196(17)30082-7 29851641

[9] Dixon J, Field J, Gibson E, Martin N. Curriculum content for Environmental Sustainability in Dentistry. J Dent. 2024;147:105021. 38679135 10.1016/j.jdent.2024.105021

[10] Public Health England. Carbon modelling within dentistry: Towards a sustainable future. London: Public Health England; 2018.

[11] Bamedhaf O, Salman H, Tegginmani SA, Guraya SS. Environmental sustainability in the dental curriculum: a scoping review. BMC Med Educ. 2025;25:844. 40474144 10.1186/s12909-025-07441-y PMC12143048

[12] Hackley DM, Luca J. Sustainability in dentistry: an overview for oral healthcare team members. J Calif Dent Assoc. 2024;52:2422150.

[13] Martin N, Sheppard M, Gorasia G, Arora P, Cooper M, Mulligan S. Drivers, opportunities and best practice for sustainable dental practice - a scoping literature review: sustainable dental practice: drivers and opportunities. J Dent. 2021;112:103737. 10.1016/j.jdent.2021.103737 34182061

[14] Duane B, Dixon J, Ambibola G, Aldana C, Couglan J, Henao D, et al. Embedding environmental sustainability within the modern dental curriculum- exploring current practice and developing a shared understanding. Eur J Dent Educ. 2021;25:541–9. 33230919 10.1111/eje.12631

[15] Field J, Martin N, Duane B, Vital S, Mulligan S, Livny A, et al. Embedding environmental sustainability within oral health professional curricula—Recommendations for teaching and assessment of learning outcomes. Eur J Dent Educ. 2023;27:650–61. 36121067 10.1111/eje.12852

[16] General Dental Council. The Safe Practitioner: a framework of behaviours and outcomes for dental professional education: consultation outcome report. 2023. Available from: https://www.gdc-uk.org/docs/default-source/education-and-cpd/safe-practitoner/spf-dentist.pdf?sfvrsn=c198211d_5 [Accessed 12 Jan 2025].

[17] Federation FWD. Sustainability in dentistry: adopted by the FDI general assembly: August 2017, Madrid, Spain. Int Dent J. 2025;68:10. 10.1111/idj.12369 PMC9378915 29363120

[18] Dixon J, Martin N, Field J. Current practice, barriers and drivers to embedding environmental sustainability in undergraduate dental schools in the UK and Ireland. Br Dent J. 2024;237:723–8. 39516621 10.1038/s41415-024-8011-6 PMC11549032

[19] Dixon J, Tubert-Jeannin S, Davies J, van Harten M, Roger-Leroi V, Vital S, et al. O-Health-Edu: a viewpoint into the current state of oral health professional education in Europe: Part 2: Curriculum structure, facilities, staffing and quality assurance. Eur J Dent Educ. 2024;28:607–20. 38258340 10.1111/eje.12987

[20] Dixon J, Baird HM, Field J, Martin N. Longitudinal integration of environmental sustainability in the dental curriculum: assessing changes in student awareness, attitudes and knowledge. J Dent. 2025;156:105710. 40132787 10.1016/j.jdent.2025.105710

[21] Joury E, Lee J, Parchure A, Mortimer F, Park S, Pine C, et al. Exploring environmental sustainability in UK and US dental curricula and related barriers and enablers: a cross-sectional survey in two dental schools. Br Dent J. 2021;230:605–10. 33990748 10.1038/s41415-021-2942-y

[22] Satterlee WG, Eggers RG, Grimes DA. Effective medical education: insights from the Cochrane Library. Obstet Gynecol Surv. 2008;63:329–33. 18419832 10.1097/OGX.0b013e31816ff661

[23] Forsetlund L, O’Brien MA, Forsén L, Mwai L, Reinar LM, Okwen MP, et al. Continuing education meetings and workshops: effects on professional practice and healthcare outcomes. Cochrane Database Syst Rev. 2021;9:CD003030. 34523128 10.1002/14651858.CD003030.pub3 PMC8441047

[24] Elliott E, Sharma S, Omar A, Hurst D, Marshall C, Blair A, et al. A multi-centre early evaluation of the effectiveness of workshop teaching to improve the confidence of UK and Irish dental students when addressing patient mental health. Br Dent J. 2021:1–6. 10.1038/s41415-021-3613-8. 10.1038/s41415-021-3613-8 PMC8609992 34815478

[25] Biggs J, Tang C. Teaching for Quality Learning at University: What the Student Does. 3^rd^ ed. Society for Research into Higher Education & Open University Press; 2003.

[26] McArthur J. Rethinking authentic assessment: work, well-being, and society. High Educ. 2023;85:85-101. 10.1007/s10734-022-00822-y. 10.1007/s10734-022-00822-y PMC8853385 35194229

[27] Queen Mary University of London. Promote sustainability. Queen Mary Academy. Available from: https://www.qmul.ac.uk/queenmaryacademy/educators/resources/graduate-attributes/promote-sustainability/ [Accessed 12 Jan 2025].

[28] Bouton ME. Why behaviour change is difficult to sustain. Prev Med. 2014;68:29–36. 24937649 10.1016/j.ypmed.2014.06.010 PMC4287360

[29] Artino AR Jr. Academic self-efficacy: from educational theory to instructional practice. Perspect Med Educ. 2012;1:76–85. 23316462 10.1007/s40037-012-0012-5 PMC3540350

[30] Michie S, van Stralen MM, West R. The behaviour change wheel: a new method for characterising and designing behaviour change interventions. Implement Sci. 2011;6:42. 10.1186/1748-5908-6-42. 21513547 10.1186/1748-5908-6-42 PMC3096582

